# A parameter adaptive method for state of charge estimation of lithium-ion batteries with an improved extended Kalman filter

**DOI:** 10.1038/s41598-021-84729-1

**Published:** 2021-03-11

**Authors:** Shichun Yang, Sida Zhou, Yang Hua, Xinan Zhou, Xinhua Liu, Yuwei Pan, Heping Ling, Billy Wu

**Affiliations:** 1 School of Transportation Science and Engineering, Beihang University, Beijing, China; 2 BYD Auto Industry Co., Ltd, Shenzhen, 518118 China; 3 Dyson School of Design Engineering, Imperial College London, London, UK

**Keywords:** Batteries, Electrical and electronic engineering

## Abstract

An accurate state of charge (SOC) estimation in battery management systems (BMS) is of crucial importance to guarantee the safe and effective operation of automotive batteries. However, the BMS consistently suffers from inaccuracy of SOC estimation. Herein, we propose a SOC estimation approach with both high accuracy and robustness based on an improved extended Kalman filter (IEKF). An equivalent circuit model is established, and the simulated annealing-particle swarm optimization (SA-PSO) algorithm is used for offline parameter identification. Furthermore, improvements have been made with noise adaptation, a fading filter and a linear-nonlinear filtering based on the traditional EKF method, and rigorous mathematical proof has been carried out accordingly. To deal with model mismatch, online parameter identification is achieved by a dual Kalman filter. Finally, various experiments are performed to validate the proposed IEKF. Experimental results show that the IEKF algorithm can reduce the error to 2.94% under dynamic stress test conditions, and robustness analysis is verified with noise interference, hence demonstrating its practicability for extending to state estimation of battery packs applied in real-world operating conditions.

## Introduction

Due to the global energy crisis and environmental pollution issues, electric vehicles (EVs) are being developed as alternatives to traditional internal combustion engine powered vehicles^1,2^. As the main energy storage system for EVs, battery packs are made of numerous lithium-ion batteries (LIBs), with close monitoring of the battery states essential to maintaining safe and efficient operation^3^, which emphases the importance of the battery management system (BMS). The BMS serves a number of purposes including the determination of the remaining energy, estimating state of available power, voltage monitoring, cell balancing and lifetime predictions^4^.

The state of charge (SOC) characterises the available capacity of a cell and its estimation is one of the basic but vital functions for a BMS. Accurate SOC estimation can thus help the BMS with cell balancing in packs and avoid over-charging and over-discharging of cells which will damage the batteries^5^. Unfortunately, SOC is difficult to measure directly, with the accuracy affected by factors such as temperature and degradation, resulting in a complicated control problem.

SOC estimation methods can be divided into definition-based and model-based approaches which have been applied to a range of different battery chemistries^6^. The Ampere-hour counting (AHC) method, also known as Coulomb counting method, is one of the basic methods of defining the SOC which is shown in Eq. (1):

1 $$SOC_{t + 1} = SOC_{t} - \frac{\eta }{{Q_{C} }}\int_{t}^{t + 1} {i_{t} dt}$$

where *η* is the charge coefficient, which is assumed to be 1 in this article, and $Q_{C}$ is the battery nominal capacity (Ah).

Online SOC estimation typically adopts the AHC method due to its adaptability for embedded systems on EVs. However, the AHC has the disadvantage of poor robustness in practical applications, especially when dealing with cumulative errors or incorrect initialization.

Model-based methods utilise mathematical models describing the batteries behaviour with varying degrees of complexity, with common forms including the equivalent circuit models (ECM), data-driven approaches, physics-based models and finite element models (FEM). ECMs are composed of circuit components including capacitors and resistors, to represent dynamic voltage characteristic^7^. Data-driven models are based on the data mining approaches, such as neural network or support vector machines (SVM), which establish black-box models without considering battery physics^8^. Furthermore, data-driven models rely on datasets for training to achieve ideal generalization which can be costly and time-consuming. Therefore, the model accuracy cannot be ensured unless sufficient data is provided over all permutations of operation. Physics based models describe the internal electrochemical processes such as diffusion and charge transfer, and mainly include the single particle model (SPM) and pseudo-two-dimensional (P2D) model^9^. FEMs are often used to describe 3D heterogeneities in cell thermal performance with large computationally cost.

Therefore, FEMs and data-based methods are rarely used in embedded system of EVs due to the large amount of calculation needed. However, real world implementation of state-estimation algorithms is often plagued with signal noise which can result in cumulative errors. Motivated by this, Kalman filters are frequently used with models to accurately estimate battery status such as SOC in the presence of signal noise^10^.

A Kalman filter itself, is an efficient filtering algorithm proposed for tracking the state of linear systems in Gaussian noise environments. However, when applied to LIBs which exhibit hysteresis effects and strong nonlinearities during charging/discharging conditions, the performance of Kalman filter is often limited^11^. Therefore, improved methods have been put forward to solve this issue. This includes the extended Kalman filter (EKF) which uses Taylor series to achieve system linearization^12^ to study SOC estimation^13–15^, parameter estimation^16^, and residual life estimation^17,18^.

However, the traditional EKF method or other similar algorithms rarely consider the complex coupling effect of cell parameters. As a result, more adaptive approaches are urgently needed to avoid parameters drifting over the lifetime of operation. Efforts to address this include Wang et al.^19^ who introduced an EKF method coupled with simultaneously identified parameters. Zheng et al.^13^ investigated SOC estimation with an EKF and particle filter (PF) based approach with a SOC differential voltage model. Furthermore, Wang et al.^20^ adopted a dual Kalman filter (DKF) based approach to deal with the differences in charging and discharging behaviour. However, despite these efforts there are still challenges associated with accurate SOC estimation and more coupled methods should be considered, especially for applications in EV embedded systems.

To address the issues of SOC estimation of LIBs, this paper proposes an improved EKF (IEKF) algorithm based on ECM, which has a precise filtering system and time-varying battery model parameters, to effectively improve the model accuracy, robustness and convergence of the algorithm. The research contributes to the operational applications of the family of Kalman filters on the battery management system. The improvements take the practical issues into consideration; thus, the targeted optimizations aim to provide possible solutions for actual applications.

This work is organized as follows. Firstly, the Thevenin equivalent circuit model is introduced and model parameters are identified offline by using simulated annealing-particle swarm optimization (SA-PSO) method. Then the IEKF algorithm is presented with rigorous proof addressing noise adaptation and fading filtering. Furthermore, a method for online parameter identification is illustrated based on DKF. Finally, experimental results employing a designed workbench for algorithm validation is discussed and compared with the EKF, including comparisons of the precision, robustness and convergence.

## Lithium-ion battery modeling

### Thevenin equivalent circuit model

**Figure 1:**
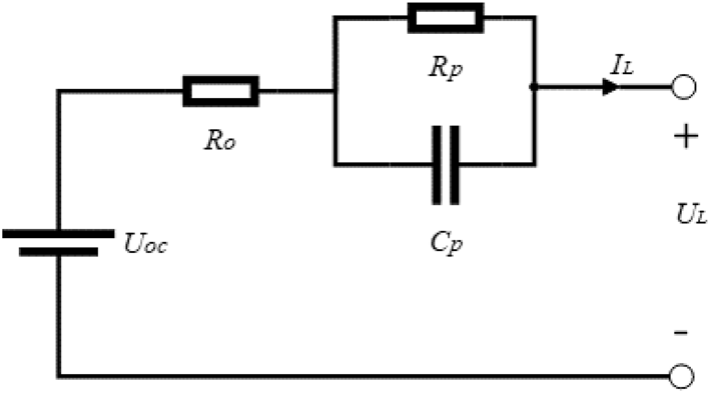
Thevenin equivalent circuit model.

Various battery models have been discussed in “Introduction”. Considering the characteristics of several types of battery models, ECMs are often adopted^21^. Thus, a representative Thevenin model used in this article is shown in Fig. 1.

According to Fig. 1, the Thevenin ECM can be described by Eq. (2) which are derived from Kirchhoff Voltage Law (KVL):

2 $$\left\{ \begin{gathered} U_{o} = I_{L} R_{o} \hfill \\ \dot{U}_{p} = \frac{{I_{L} }}{{C_{p} }} - \frac{{U_{p} }}{{R_{p} C_{p} }} \hfill \\ U_{L} = U_{OC} - U_{p} - U_{o} \hfill \\ \end{gathered} \right.$$

The Thevenin model consists of ideal voltage source representing open circuit voltage (OCV, UOC), the ohmic resistance *R*_*o*_ and a RC network. *R*_*p*_ and *C*_*p*_ are the polarization resistance and capacitance respectively. *U*_*L*_ is the terminal voltage, *I*_*L*_ is the current (assumed positive for charge, negative for discharge). *U*_*p*_ describes the polarization voltage over the RC network.

For Eq. (1) and Eq. (2), a Laplace transform is used to establish a state-space equation for the Thevenin ECM, shown as Eq. (3):

3 $$\left[ \begin{gathered} SOC_{k + 1} \hfill \\ U_{P,k + 1} \hfill \\ \end{gathered} \right] = \left[ {\begin{array}{*{20}c} 1 & 0 \\ 0 & {e^{{ - T_{s} /R_{p} C_{p} }} } \\ \end{array} } \right]\left[ \begin{gathered} SOC_{k} \hfill \\ U_{P,k} \hfill \\ \end{gathered} \right]+ \left[ {\begin{array}{*{20}c} { - \frac{{\eta T_{s} }}{{Q_{c} }}} \\ {R_{p} (1 - e^{{ - T_{s} /R_{p} C_{p} }} )} \\ \end{array} } \right]I_{k} + {\varvec{w}}_{k}$$

where *T*_*s*_ is the sampling interval, ***w***_*k*_ is the processing noise.

The systematic observation equation is shown in Eq. (4):

4 $$U_{L,k} = U_{ocv} - U_{p} - R_{o} I_{L,k} + v_{k}$$

where: *v*_*k*_ is the observation noise.

For the established state-space model, the parameters to be identified consist of *C*_*p*_, *R*_*o*_ and *R*_*p*_. Classical methods for parameter identification are divided into offline and online methods. Since the parameters of the battery are greatly affected by the SOC, state of health (SOH) (representing the battery remaining life) as well as temperature and charging/discharging current, it is difficult to obtain accuracy parameters if relying on interpolated values which were acquired from simple offline parameter identification tests. Therefore, in this article, online parameter identification is applied to obtain better precision of the battery parameters, which is called DKF as shown in “IEKF and parameter adaptation”. To speed up the fitting process, a SA-PSO method has been developed for offline parameter identification, which can be used as the initial value of online estimation and implemented for model correction.

### Offline parameter identification

Although online parameter identification can effectively improve the accuracy of the ECMs, the algorithm used will degenerate if initialization of parameters deviates too much from the true value, and result in a lower convergence speed because the parameters correction relies on observation values. Therefore, a SA-PSO method has been adopted for identifying the parameters offline as the initial value of online identification.

The SA algorithm originates from the industrial annealing process and has been applied for various scenarios^22^. For each feasible solution generated, the value of the fitness function (marked as f(x)) is calculated and the optimal solution is selected according to the Metropolis principle which is a type of resampling method. In this article, the new state will be generated by a normal distribution. When the system status varies from *x*_*0*_ to *x,* there is a probability:

5 $$p = \exp ( - \frac{{E_{x} - E_{{x_{0} }} }}{T})$$

where *P* is the probability of the new state being acceptable; $E$ is the energy of system, which can be abstract as the fitness function and *T* is the annealing temperature. In this article, we use the root mean squared error (RMSE) between the simulated voltage and the real voltage as the fitness function.

If *E*_*x0*_ < *E*_*x*_, the new status will be accepted; otherwise, the new status will be discarded by the probability:

6 $$p(x \to x0) = \left\{\begin{array}{*{20}l} 1, & E_{x0} < E_{x} \\ \exp ( - \frac{{E_{x0} - E_{x} }}{T}), & E_{x0} \ge E_{x} \\ \end{array} \right.$$

During each annealing temperature, *L* times iteration will be executed. After the iterative process is completed, *T* will be updated as Eq. (7) the maximum posteriori estimation proposed

7 $$T_{new} = K*T$$

where *K* is the annealing rate. The performance of SA is significantly influenced by the annealing rate and a fully slow annealing is appreciated for the best solution searching. Therefore, *K* is 0.99 in this article.

The SA algorithm can search the global-best solution; however, the capability of a global search relies on a sufficiently high temperature initialization and a sufficiently slow cooling process. Therefore, the SA algorithm converges slowly, but can be optimize by coupling this with an intelligent optimization method^23^.

The PSO algorithm belongs to the group intelligent optimization methods and is derived from the study of bird predation behaviors. PSO, including its implement, has been widely used for parameter identification^24,25^. In the PSO algorithm, each potential solution can be abstracted as a particle in state-space with each particle having a position and velocity. All particles simultaneously search for the individual-best solution and group-best solution in the given state-space. The position-speed update equation is shown as Eq. (8).

8 $${\mathbf{v}}_{k + 1} = \omega {\mathbf{v}}_{k} + c_{1} r_{1} [{\mathbf{p}}_{k} - {\mathbf{x}}_{k} ] + c_{2} r_{2} [{\mathbf{g}}_{k} - {\mathbf{x}}_{k} ]$$

where ***ω*** is the inertia weight; *c*_1_ and *c*_2_ are learning factors; *r*_1_ and *r*_2_ are random numbers between 0 and 1; ***v*** is the velocity of particle; ***x*** is the current solution;* p* is the individual-best solution and* g* is the group-best solution. In this article, we assume that *c*_1_ = *c*_2_ = 1.5, and *ω* = 0.8.

The position can thus be updated by Eq. (9):

9 $${\mathbf{x}}_{k + 1} = {\mathbf{x}}_{k} + {\mathbf{v}}_{k}$$

The PSO algorithm has a high convergence speed, but it is intended to converge to a local-best solution.

To optimize the SA algorithm and the PSO algorithm, a coupled SA-PSO algorithm is proposed to speed up convergence speed and avoid the algorithm falling into local optimum. The specific implementation manner can thus be depicted as follows:

Step 1: set the initial annealing temperature, and perform a Monte Carlo search at each temperature to find the global equilibrium status.

Step 2: when each particle is iteratively updated in the particle swarm algorithm, the Metropolis criterion is adopted to accept the new particle status.

Step 3: loop at each annealing temperature until the number of iterations reaches the maximum or a satisfactory convergence is achieved.

**Figure 2:**
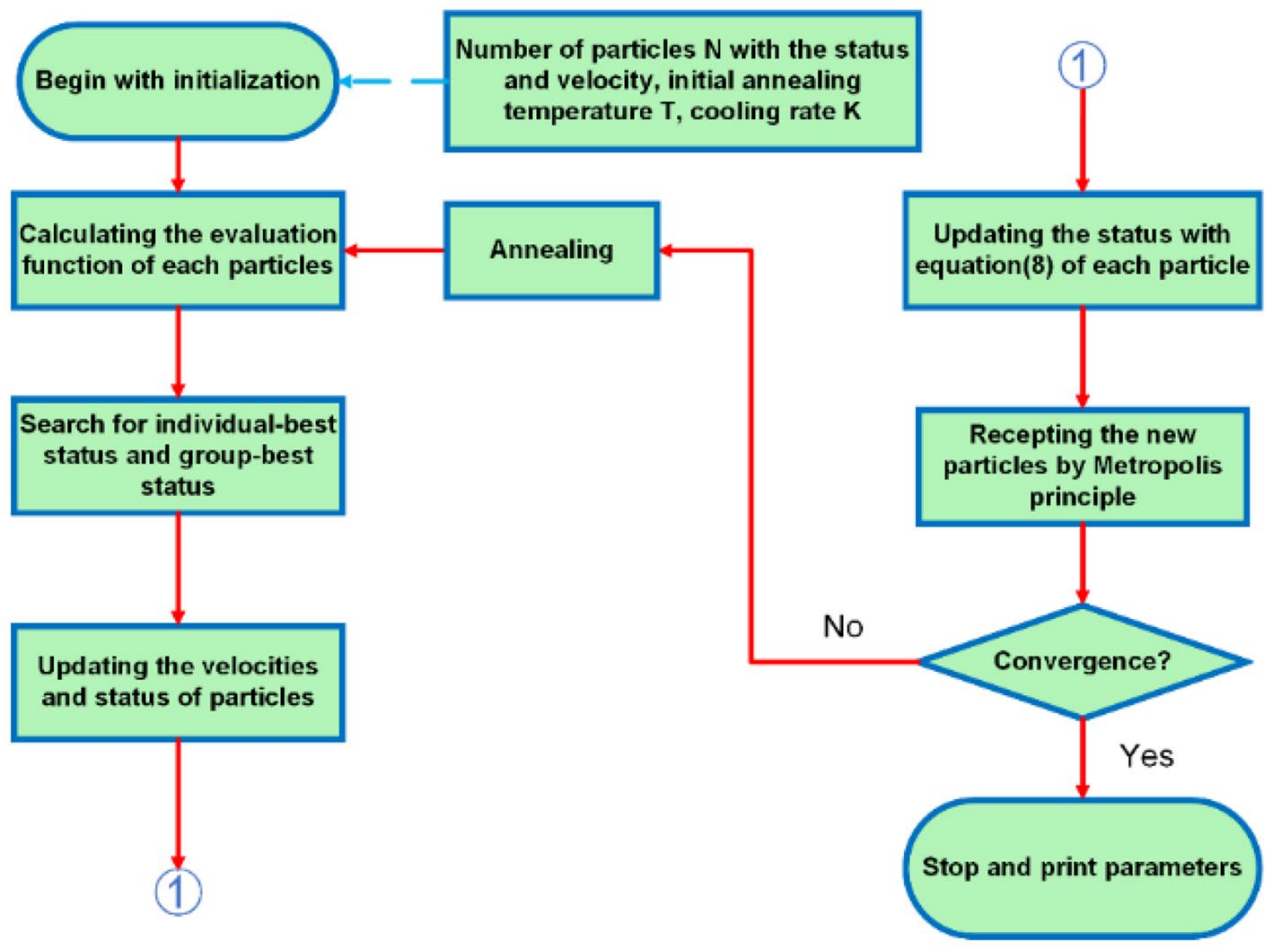
Procedure of the proposed PSO-SA method.

Using this method, the algorithm not only avoids local optimum, but also guarantees the Monte Carlo simulation process of the annealing algorithm to ensure the ability to search the global parameter space. The SA-PSO algorithm can be depicted as Fig. 2.

**Figure 3:**
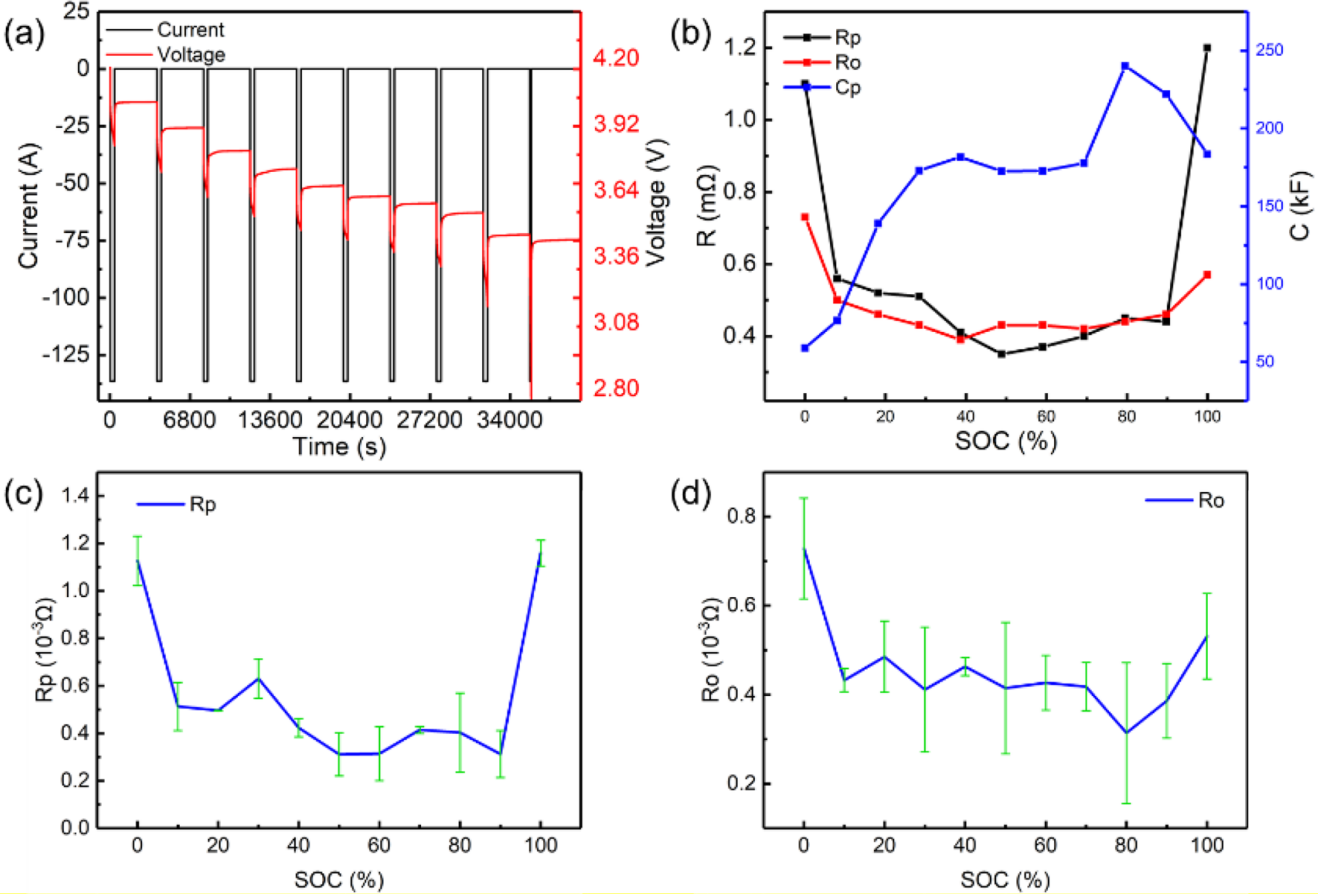
The experiment and results of offline parameter identification based on SA-PSO: **(a)** experimental measurement; **(b)** the result of parameter identification; **(c)** the error bar of identified Rp; **(d)** the error bar of identified Ro.

Pulse discharge experiments were carried out to validate the algorithm. The experiment results are shown in Fig. 3a. According to Eq. (2), the system has zero-input in the voltage rebound phase, which can be used to fit *R*_*p*_ and *C*_*p*_ values, and the voltage step at the end of the pulse discharge can be used to fit *R*_*o*_. The offline parameter identification fitting results are shown in the Fig. 3b. Moreover, the error bars of Ro and Rp are presented as Fig. 3c,d, which are demonstrated based on the multi-times simulation results. Considering the error analysis based on error bars, the adopted models generally predict the battery characteristics and can be applied for states estimation.

## IEKF and parameter adaptation

### Traditional EKF

The classical Kalman filter performs poorly in state estimation problems when dealing with nonlinear systems. The main cause is that there are nonlinear integral equations when the state and observers update. Based on Bayesian filtering theory, nonlinear systems that satisfy Markov characteristics can be depicted as Eq. (10):

10 $$\left\{ \begin{gathered} {\mathbf{x}} \sim p({\mathbf{x}}_{k} |{\mathbf{x}}_{k - 1} ) \hfill \\ z \sim p(z_{k} |{\mathbf{x}}_{k} ) \hfill \\ \end{gathered} \right.$$

where *p* is the probability of state transfer, and ***x ***is the system state, and *z* is the system observation.

The priori information (one-step prediction equation) can be defined as Eq. (11) and state updating equation as Eq. (12):

11 $$p({\mathbf{x}}_{k} |z_{k - 1} ) = \int {p({\mathbf{x}}_{k} |{\mathbf{x}}_{k - 1} )p({\mathbf{x}}_{k - 1} |z_{k - 1} )d{\mathbf{x}}_{k - 1} }$$

12 $$p({\varvec{x}}_{k} |z_{k} ) = \frac{{p(z_{k} |{\varvec{x}}_{k} )p({\varvec{x}}_{k} |z_{k - 1} )}}{{p(z_{k} |z_{k - 1} )}}$$

13 $$p(z_{k} |z_{k - 1} ) = \int {p(z_{k} |{\mathbf{x}}_{k} )p({\mathbf{x}}_{k} |z_{k - 1} )d{\mathbf{x}}_{k} }$$

where $p({\mathbf{x}}_{k} |z_{k - 1} )$ is priori information,$\frac{{p(z_{k} |{\mathbf{x}}_{k} )}}{{p(z_{k} |z_{k - 1} )}}$ is maximum likelihood function.

Equation (11) and Eq. (12) represent the prediction and updating process respectively. The classical linear Kalman filter has difficulty in calculating Eq. (11) and Eq. (13) precisely. Therefore, some improvement is needed to improve the algorithm performance. Here, a Taylor series expansion of the nonlinear equations is used to achieve linearization, which is called EKF. The EKF has first-order polynomial precision, for which it discards the quadratic term and high order terms (Table 1).

**Table 1:** The comparisons between diverse Kalman filters.

Item	Advantages	Disadvantages
Kalman filter	Low calculation and great precision with linear equations	Worse precision with non-linear equations
Extended Kalman filter	Low calculation and well precision with non-linear equations	Difficulty for linearizing the equations
Unscented Kalman filter	Medium-calculation and well precision with non-linear equations	Unpredictable factors inside equations
Cubature Kalman filter	Great precision with non-linear equations	Much calculations

For the nonlinear system described in Eq. (14):

14 $$\left\{ \begin{gathered} {\mathbf{x}}_{k} = {\mathbf{f}}({\mathbf{x}}_{k - 1} ,{\mathbf{u}}_{k} ,{\mathbf{w}}_{k} ) \hfill \\ {\mathbf{z}}_{k} = {\mathbf{h}}({\mathbf{x}}_{k} ,{\mathbf{v}}_{k} ) \hfill \\ \end{gathered} \right.$$

where *u*_*k*_ indicates the input/control amount at time *k*.

Extended Kalman filter can be summarized as Table 2.

**Table 2:** Extended Kalman filter algorithm.

**Initialization**	
	$${\hat{\mathbf{x}}}_{0} = E[{\mathbf{x}}_{0} ]$$
	$${\mathbf{P}}_{0} { = }E[({\mathbf{x}} - {\hat{\mathbf{x}}}_{0} )({\mathbf{x}} - {\hat{\mathbf{x}}}_{0} )^{T} ]$$
**Prediction module**	
Status prediction	$\hat{x}_{k|k - 1} = f(\hat{x}_{k - 1} ,u_{k} ,w_{k - 1} )$
Covariance matrix prediction	${\mathbf{P}}_{k|k - 1} = {\mathbf{A}}_{k - 1} {\mathbf{P}}_{k} {\mathbf{A}}_{k - 1}^{T} + {\mathbf{Q}}_{k - 1}$
where:	$${\mathbf{A}}_{k - 1} = \left. {\left[ {\begin{array}{*{20}c} {\frac{{\partial f_{1} }}{{\partial x_{1} }}} & \ldots & {\frac{{\partial f_{1} }}{{\partial x_{n} }}} \\ \vdots & \ddots & \vdots \\ {\frac{{\partial f_{m} }}{{\partial x_{1} }}} & \ldots & {\frac{{\partial f_{m} }}{{\partial x_{n} }}} \\ \end{array} } \right]} \right|_{{x = \hat{x}_{k - 1} }}$$
**Updating module**	
Kalman gain	${\mathbf{G}}_{k} = {\mathbf{P}}_{k|k - 1} {\mathbf{C}}_{k}^{T} ({\mathbf{C}}_{k} {\mathbf{P}}_{k|k - 1} {\mathbf{C}}_{k}^{T} + {\mathbf{R}}_{k - 1} )^{ - 1}$
where:	$${\mathbf{C}} = \left. {\left[ {\begin{array}{*{20}c} {\frac{{\partial g_{1} }}{{\partial x_{1} }}} & \ldots & {\frac{{\partial g_{1} }}{{\partial x_{n} }}} \\ \vdots & \ddots & \vdots \\ {\frac{{\partial g_{m} }}{{\partial x_{1} }}} & \ldots & {\frac{{\partial g_{m} }}{{\partial x_{n} }}} \\ \end{array} } \right]} \right|_{{x = \hat{x}_{k|k - 1} }}$$
Status update	${\hat{\mathbf{x}}}_{k} = {\hat{\mathbf{x}}}_{k|k - 1} + {\mathbf{G}}_{k} (z_{k} - {\mathbf{C}}_{k} {\hat{\mathbf{x}}}_{k|k - 1} )$
Covariance matrix update	${\mathbf{P}}_{k} = ({\mathbf{I}} - {\mathbf{G}}_{k} {\mathbf{C}}_{k} ){\mathbf{P}}_{k|k - 1}$

The linearization process can be expressed as follows:

$${\mathbf{A}} = \left[ {\begin{array}{*{20}c} {\frac{{\partial f_{1} }}{{\partial x_{1} }}} & \ldots & {\frac{{\partial f_{1} }}{{\partial x_{n} }}} \\ \vdots & \ddots & \vdots \\ {\frac{{\partial f_{m} }}{{\partial x_{1} }}} & \ldots & {\frac{{\partial f_{m} }}{{\partial x_{n} }}} \\ \end{array} } \right] \quad {\mathbf{B}} = \left[ {\begin{array}{*{20}c} {\frac{{\partial f_{1} }}{{\partial u_{1} }}} & \ldots & {\frac{{\partial f_{1} }}{{\partial u_{n} }}} \\ \vdots & \ddots & \vdots \\ {\frac{{\partial f_{m} }}{{\partial u_{1} }}} & \ldots & {\frac{{\partial f_{m} }}{{\partial u_{n} }}} \\ \end{array} } \right]$$

$${\mathbf{C}} = \left[ {\begin{array}{*{20}c} {\frac{{\partial g_{1} }}{{\partial x_{1} }}} & \ldots & {\frac{{\partial g_{1} }}{{\partial x_{n} }}} \\ \vdots & \ddots & \vdots \\ {\frac{{\partial g_{m} }}{{\partial x_{1} }}} & \ldots & {\frac{{\partial g_{m} }}{{\partial x_{n} }}} \\ \end{array} } \right]$$

### Improved EKF

Although the EKF improves the ability of the algorithm to cope with nonlinear systems, more improvements are needed for complex system state tracking problems in practical applications. For real-world battery pack, the system noise is often non-Gaussian, such as the constant deviation of the current and voltage sensor, especially for large capacity battery packs. Additionally, the characteristics are strongly coupled with battery capacity degradation, ambient temperature and dynamic working conditions, affecting the accuracy and robustness of the SOC estimation. Therefore, some optimization is adopted to improve the EKF algorithm to impair the influences of the factors mentioned.

#### Adaptive noise

For the Kalman filter, signal noise can be separated into process noise and environmental noise. The process noise characterizes the reliability of the proposed model, and the environmental noise often originates from errors caused by the sensor and environmental disturbance during the actual measurement. Both noises have great impacts on the performance of the filtering system, mainly affecting the convergence and accuracy of the system^26^. For a converged Kalman filter, the system is considered to be converged to a stable value at an infinite moment as shown in Eq. (15)

15 $$\mathop {\lim }\limits_{k \to \infty } P_{k} = P_{const}$$

According to the covariance matrix updating the EKF, the Kalman Gain converges to a constant value along with the convergent covariance matrix. Therefore, the Kalman filter will degrade to a low-pass filter, and the Kalman Gain is only determined by system noise, which affects the precision.

On the other hand, the process noise influences convergence speed of the system. The existence of noise can assist the system to gradually converge to the true value when there is a deviation, and the convergence speed depends on the amplitude of the noise. In real operating conditions, it is generally expected that there is a larger noise in the initial stage of filtering to enhance the influence of correcting initial value deviation and enhancing the convergence speed of the filtering. However, when the filter is stabilized, it is ideal for the noise to be attenuated for higher accuracy. Therefore, it is of great practical significance to achieve adaptive system noise^27^. The adaptive noise can be effectively implemented with observation. The demand of data storage for recursive process is small, and with the low dimensions of the matrix ***A***,* B*,* C*, the adaptive algorithm has obvious advantage for calculation.

The adaptive algorithm based on the maximum posteriori estimation proposed for discrete systems can be characterized as Eq. (16) and (17)^28^:

16 $$\left\{ \begin{gathered} \hat{w}_{k} = (1 - d_{k} )\hat{w}_{k - 1} + d_{k} (\hat{x}_{k} - (A\hat{x}_{k - 1} + Bu_{k} )) \hfill \\ \hat{v}_{k} = (1 - d_{k} )\hat{v}_{k - 1} + d_{k} (z_{k} - C_{k} x_{k} ) \hfill \\ \end{gathered} \right.$$

17 $$\left\{ \begin{array}{*{20}l} \hat{Q}_{k} = (1 - d_{k} )\hat{Q}_{k - 1} \\ + d_{k} (G_{k} \varepsilon \varepsilon^{T} G_{k}^{T} + P_{k} - A_{k - 1} P_{k|k - 1} A_{k - 1}^{T} ) \\ \hat{R}_{k} = (1 - d_{k} )\hat{w}_{k - 1} \\ + d_{k} (\varepsilon \varepsilon^{T} - C_{k} P_{k|k - 1} C_{k}^{T} ) \\ \end{array} \right.$$

where $d_{k} = (1 - b)/(1 - b^{k} )$, *b* is a constant between 0 and 1, $\varepsilon = z_{k} - C_{k} x_{k}$.

#### Fading filter

The fading filter is a method to enhance the system's ability for utilizing observation measurement to optimize divergence phenomenon when filtering. Due to the rounding error when calculating with computers, the covariance matrix may not be positive during the iterative process, leading to the filtering system oscillating or diverging. In addition, when the system model is severely mismatched, the correction of the system can only be achieved with observation. Therefore, enhancing the observation proportion when updating the status can improve the stability and robustness of the algorithm.

Classical EKF algorithms assume that the total filtering time domain is *N*, for the arbitrary time *k* in the time domain, thus we have:

18 $$P_{k|k - 1}^{N} = A_{k - 1} P_{k - 1}^{N} A_{k - 1}^{T} + Q_{k - 1}$$

Take *s* as the fading factor, and multiply both sides of the Eq. (19) by *s*^-(*N-k*)^:

19 $$s^{ - (N - k)} P_{k|k - 1}^{N} = A_{k - 1} (s^{ - (N - k)} P_{k - 1}^{N} )A_{k - 1}^{T} + Q_{k - 1}$$

Note:

20 $$P_{k|k - 1}^{N*} = s^{ - (N - k)} P_{k|k - 1}^{N}$$

21 $$P_{k - 1}^{N*} = s^{ - (N - (k - 1))} P_{k - 1}^{N}$$

Then Eq. (19) can be expressed as Eq. (22):

22 $$P_{k|k - 1}^{N*} = A_{k - 1} (sP_{k - 1}^{N*} )A_{k - 1}^{T} + Q_{k - 1}$$

Equation (18) can be replaced by Eq. (22) to optimize the covariance matrix updating, and the covariance matrix has been enlarged by *s* times compared with the original equation which reveals that the utilization ability for measurement has been enhanced.

The fading factor of Eq. (22) can select a constant larger than 1, and can also be adaptively updated according to the degree of system model mismatch Moreover, the error bars].

#### Linear–nonlinear filter

When the EKF is improved by using several solutions shown as “IEKF and parameter adaptation”, the cost of calculation needed for the algorithm obviously increases, making it difficult to realize fast estimation. Therefore, the linear–nonlinear (L–N) filter is used for solving the issues that the algorithm is too complex.

Noting that the first-order ECM has the following characteristics:

1. State update process is a linear process.

23 $${\mathbf{x}}(k + 1) = {\mathbf{Ax}}(k) + {\mathbf{w}}(k)$$

2. Observation process is non-linear process.

24 $${\mathbf{y}}(k) = f({\mathbf{x}}(k),{\mathbf{w}}(k))$$

The nonlinear proportion is concentrated in the observation process. Therefore, it is considered that the state update portion is separately processed from the observation portion. Classical Kalman filtering has higher precision when dealing with linear systems accompany little amount of calculation. However, while the EKF can response to nonlinear systems, the accuracy is similar to that of classical Kalman filtering when tackling linear systems, whereas a lot of computing power is wasted. Therefore, for the special linear-nonlinear system mentioned above, the L-N filter is used to solve the problem. The classical Kalman filter method is adopted for the status updating process, and the EKF is applied for the observation process, which effectively reduces the calculation amount required by the algorithm without affecting the accuracy of estimation.

### Summarization

In “IEKF and parameter adaptation”, several solutions are introduced for dealing with the filtering noise and divergence problems. The L–N filter method is used to reduce the amount of calculation. The IEKF algorithm adopted can be described as Table 3:

**Table 3:** Improved EKF algorithm.

**Initialization**	
	$${\hat{\mathbf{x}}}_{0} = E[{\mathbf{x}}_{0} ]$$
	${\mathbf{P}}_{0} { = }E[({\mathbf{x}} - {\hat{\mathbf{x}}}_{0} )({\mathbf{x}} - {\hat{\mathbf{x}}}_{0} )^{T} ]$ ${\hat{\mathbf{w}}}_{0} = E({\mathbf{w}}_{0} )$ ${\hat{\mathbf{v}}}_{o} = E({\mathbf{v}}_{0} )$ $s = s_{0}$
**Prediction**	
Status prediction	${\hat{\mathbf{x}}}_{k|k - 1} = {\mathbf{A}{\hat{\mathbf x}}}_{k - 1} { + }{\mathbf{B}}u_{k} { + }{\mathbf{w}}_{{k{ - }1}}$
Covariance matrix prediction	${\mathbf{P}}_{k|k - 1} = {\mathbf{A}}_{k - 1} (s \cdot {\mathbf{P}}_{k} ){\mathbf{A}}_{k - 1}^{T} + {\mathbf{Q}}_{k - 1}$
**Updating**	
Observation	$\hat{z}_{k} = {\mathbf{g}}({\mathbf{x}}_{k} ,{\mathbf{u}}_{k} ,v_{k} )$
Kalman gain	${\mathbf{G}}_{k} = {\mathbf{P}}_{k|k - 1} {\mathbf{C}}_{k}^{T} ({\mathbf{C}}_{k} {\mathbf{P}}_{k|k - 1} {\mathbf{C}}_{k}^{T} + {\mathbf{R}}_{k - 1} )^{ - 1}$
where:	$${\mathbf{C}} = \left. {\left[ {\begin{array}{*{20}c} {\frac{{\partial g_{1} }}{{\partial x_{1} }}} & \ldots & {\frac{{\partial g_{1} }}{{\partial x_{n} }}} \\ \vdots & \ddots & \vdots \\ {\frac{{\partial g_{m} }}{{\partial x_{1} }}} & \ldots & {\frac{{\partial g_{m} }}{{\partial x_{n} }}} \\ \end{array} } \right]} \right|_{{x = \hat{x}_{k|k - 1} }}$$
Status update	${\hat{\mathbf{x}}}_{k} = {\hat{\mathbf{x}}}_{k|k - 1} + {\mathbf{G}}_{k} (z_{k} - \hat{z}_{k} )$
Covariance matrix update	${\mathbf{P}}_{k} = ({\mathbf{I}} - {\mathbf{G}}_{k} {\mathbf{C}}_{k} ){\mathbf{P}}_{k|k - 1}$
Noise adaptation	$$\left\{ \begin{gathered} {\hat{\mathbf{w}}}_{k} = (1 - d_{k} ){\hat{\mathbf{w}}}_{k - 1} \\ + d_{k} ({\hat{\mathbf{x}}}_{k} - ({\mathbf{A}{\hat{\mathbf x}}}_{k - 1} + {\mathbf{Bu}}_{k} )) \\ \hat{v}_{k} = (1 - d_{k} )\hat{v}_{k - 1} \\ + d_{k} (z_{k} - \hat{z}_{k} ) \\ \end{gathered} \right.$$
	$$\left\{ \begin{gathered} \hat{Q}_{k} = (1 - d_{k} )\hat{Q}_{k - 1} \\ + d_{k} (G_{k} \varepsilon \varepsilon^{T} G_{k}^{T} + P_{k} - A_{k - 1} P_{k|k - 1} A_{k - 1}^{T} ) \\ \hat{R}_{k} = (1 - d_{k} )\hat{w}_{k - 1} \\ + d_{k} (\varepsilon \varepsilon^{T} - C_{k} P_{k|k - 1} C_{k}^{T} \\ \end{gathered} \right.$$

### Parameter adaptation

The EKF algorithm can only track the system and depending on the observations for correction. Therefore, the EKF algorithm will degenerate into an information filter with a lower convergence rate. Furthermore, improving the model accuracy or timely modifying the model to adapt the system has higher significance for improving the convergence speed and robustness.

Online parameter identification is the main means for fitting the model to real systems. The parameters of the first-order ECM include *R*_*o*_, *R*_*p*_, *C*_*p*_. *R*_*p*_ and *C*_*p*_ and represents the battery polarization effects, used for simulating the long-term and dynamic characteristics that affect the system. Therefore, the above parameters need to be implemented in the online identification process.

The online parameter identification method can be equivalent to the application of a Dual Kalman filter (DKF)^29^. Therefore, establishing a state space model for the parameters to be identified can effectively articulate how the parameter online identification works.

Since the variation of battery parameters is usually slow during degradation, we assume that the battery parameters do not change during an iterative process. Then the state space is rewritten as Eq. (25):

25 $$\left[ {\begin{array}{*{20}c} {R_{o_k + 1} } \\ {R_{p,k + 1} } \\ {C_{p,k + 1} } \\ \end{array} } \right] = \left[ {\begin{array}{*{20}c} 1 & {} & {} \\ {} & 1 & {} \\ {} & {} & 1 \\ \end{array} } \right]\left[ {\begin{array}{*{20}c} {R_{o,k} } \\ {R_{p,k} } \\ {C_{p,k} } \\ \end{array} } \right] + w_{para,k}$$

**Figure 4:**
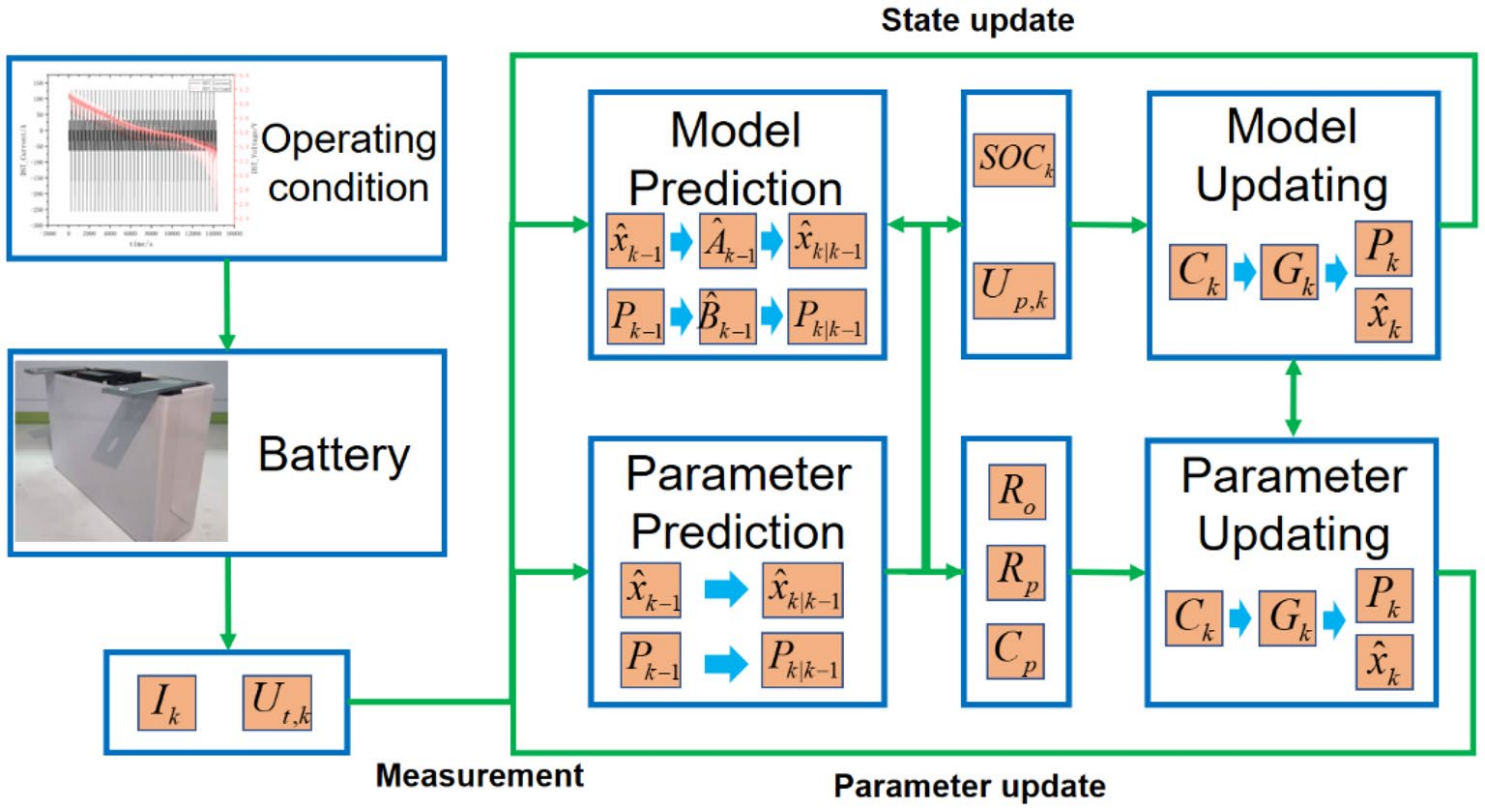
Process of online parameter identification.

This state space model has interesting features. Firstly, the state update matrix is a unit matrix, which has good positive definiteness and low computational complexity. Additionally, the observation equation of the system is the same as the battery equivalent circuit model. The observed voltage value and the polarization characteristic is affected accordingly. Therefore, the online parameter identification needs to be fed back to the prediction and update process in the ECM. Meanwhile, the SOC estimation result will affect the ability to correct the parameters which depending on observation. The adaptive parameter process is shown in Fig. 4. Considering the influence factors for filter algorithms, the selected parameters are presented in Table 4. Although the Gaussian noise is expected for ideal systems, the constant initialization is provided for predicting the non-Gaussian battery system and online parameter identification contributes to the adaptive optimizations.

**Table 4:** The selected factors of filter algorithm for SOC estimation.

Item	Value	Description
***P***	Identity matrix	The initialization of covariance matrix
***Q***	[0.01; − 0.01]	The selected processing noise
*R*	0.04	The selected observation noise
***X***_0_	[1; 0]	The initialization of state matrix

### Workbench and experiment

**Figure 5:**
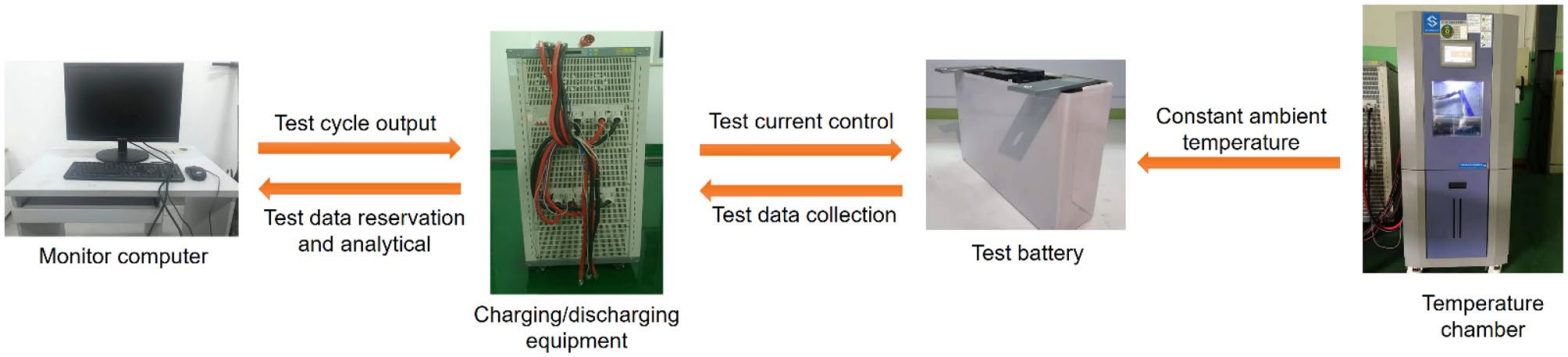
Workbench design.

To verify the validity of the algorithm, an experimental workbench is established as shown in Fig. 5, with the details illustrated in Table 5.

**Table 5:** Details of equipment.

Item	Equipment Type
Monitor computer	Ryzen 5 3500U
Battery cycler	Neware CT-4004-5V300A-NA
Test battery	BYD-130 Ah prismatic lithium-ion battery
Temperature chamber	Soyater ETE-GDW-150L environmental chamber

The experiment was verified on a BYD 130 Ah lithium-ion battery, and its specifications are shown in Table 6. The experimental results for conditions are used to verify the effectiveness and robustness of the algorithm and the verification test of this paper, including constant current test, pulse discharge test, and dynamic stress test (DST). All tests are carried out in an ambient temperature of 25 ℃.

**Table 6:** Tested battery characteristics.

Serial numbers	Parameter item	Parameter value	Unit
1	Capacity	130	Ah
2	Normal voltage	3.6	V
3	Max voltage	4.2	V
4	Min voltage	2.5	V
5	Max discharging current	300	A
6	Max charging current	250	A
7	Discharging/charging temperature range	−20 to 50	∘C
8	Cathode material	NCM	
9	Anode material	Graphite	

## Results and validation

### Algorium validation

The effectiveness of the proposed algorithm is compared to the classical EKF algorithm. Two characteristics are introduced to evaluate the algorithm, which are the maximum error (ME) and RMSE. The definitions are shown as Eqs. (26) and (27).

26 $$ME = \max \{\left| {SOC_{k} - S\hat{O}C_{k} } \right|\},k = 1,2, \cdots ,N$$

27 $$RMSE = \sqrt {\frac{1}{n}\sum\limits_{k = 1}^{N} {(SOC_{k} - S\hat{O}C_{k} )^{2} } }$$

**Figure 6:**
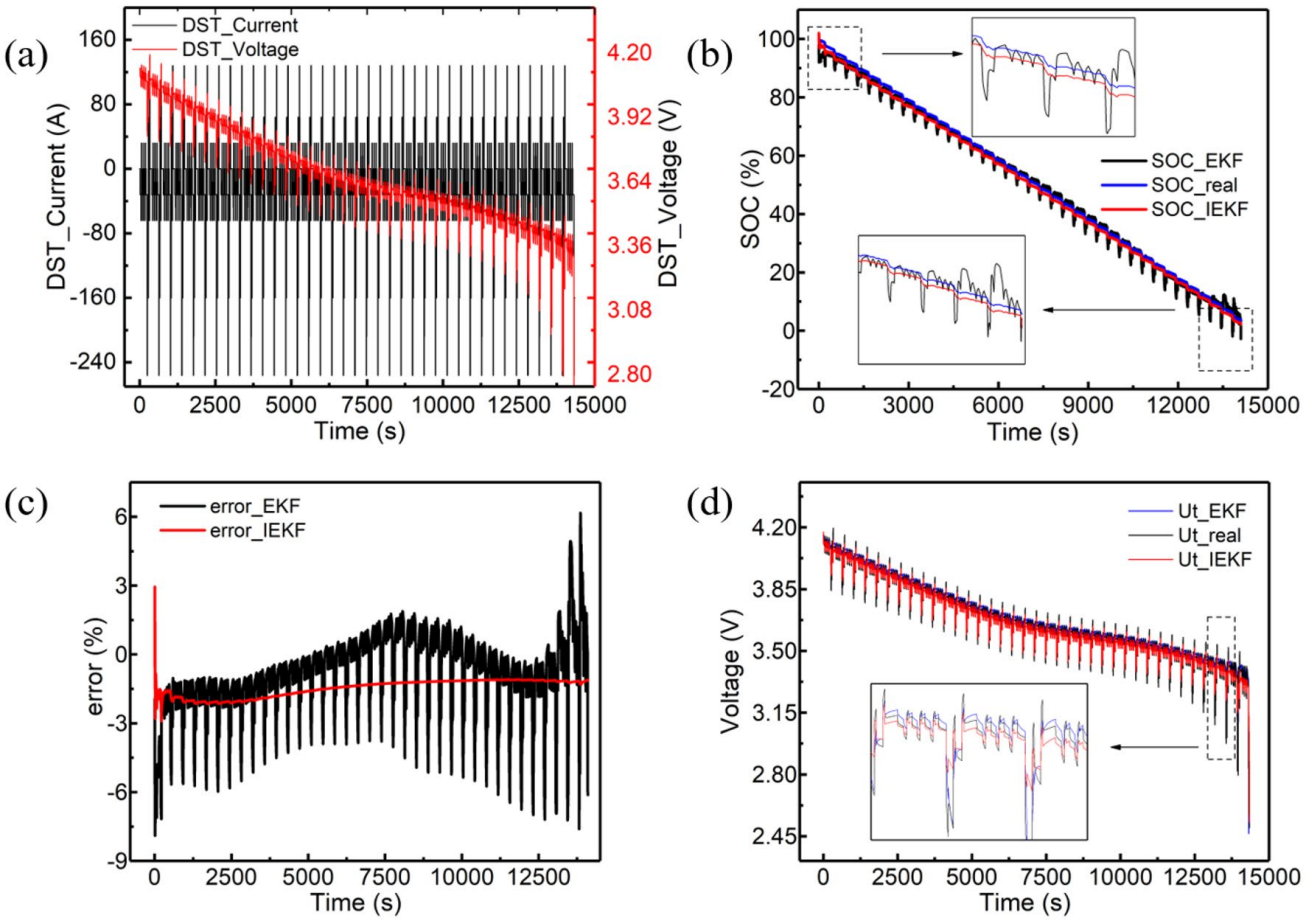
Results of IEKF and EKF under DST condition: **(a)** testing condition; **(b)** results compared with EKF; **(c)** error analysis; **(d)** The esimated voltage result profile.

The real SOC can be calculated from the measurement by using the AHC based on high precision test equipment. Compared with simulation results, the experimental results show that ME of IEKF can be reduced to 1.43% under 1C constant current discharge conditions, which is better than the 1.93% for the EKF algorithm. The RMSE of IEKF is 1.0193 × 10^–3^. Under the DST condition, the ME of IEKF slightly rises to 2.94%, and the RMSE is 2.4028 × 10^–4^. The ME of EKF rises to 6.72%. The results are shown in Table 7, which illustrate that IEKF is superior to EKF. Moreover, considering the invalidation of ECMs under low SOC ranges, the proposed method contributes to compensate the enlarged error, and the results are presented in Fig. 6b. The relatively fitted curve demonstrates the effectiveness on the correction for ECMs applying low SOC ranges.

**Table 7:** Precision comparation with IEKF and EKF.

	CC discharge	CCCV charge	Pulse discharge	DST
Error of EKF	1.93%	2.09%	2.69%	6.72%
Error of IEKF	1.43%	1.38%	1.89%	2.94%

**Figure 7:**
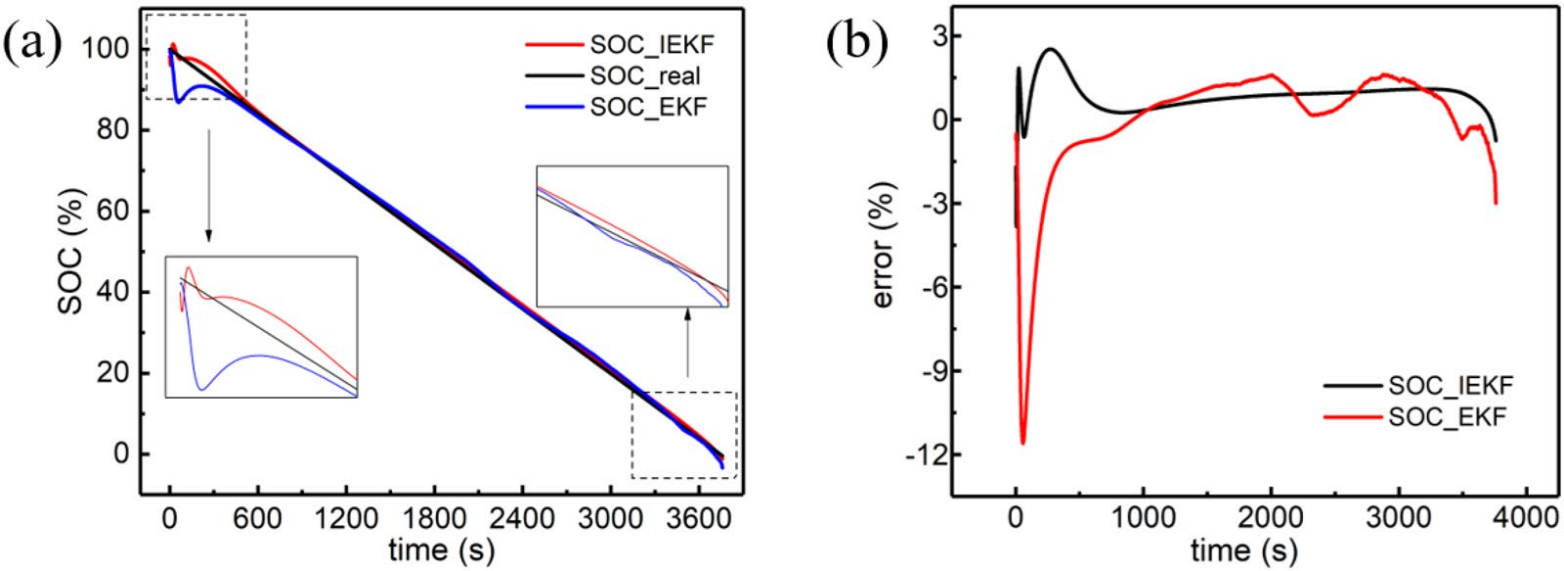
Results of IEKF used on 1C constant discharging condition: **(a)** result compared with EKF; **(b)** error analysis.

The online identification results of the model parameters can be verified according to the voltage matching degree. The results of the model matching during the DST are shown in Figs. 6, and 7.

The voltage curves show that the accuracy of the system model can be improved after using the parameter adaptive algorithm. As shown in Fig. 6d, the simulated voltage curve based on IEKF is rather similar with the real one compared against others. The results can be attributed to the effective adaptive parameter identification, and the effective correction also contributes to the result.

**Figure 8:**
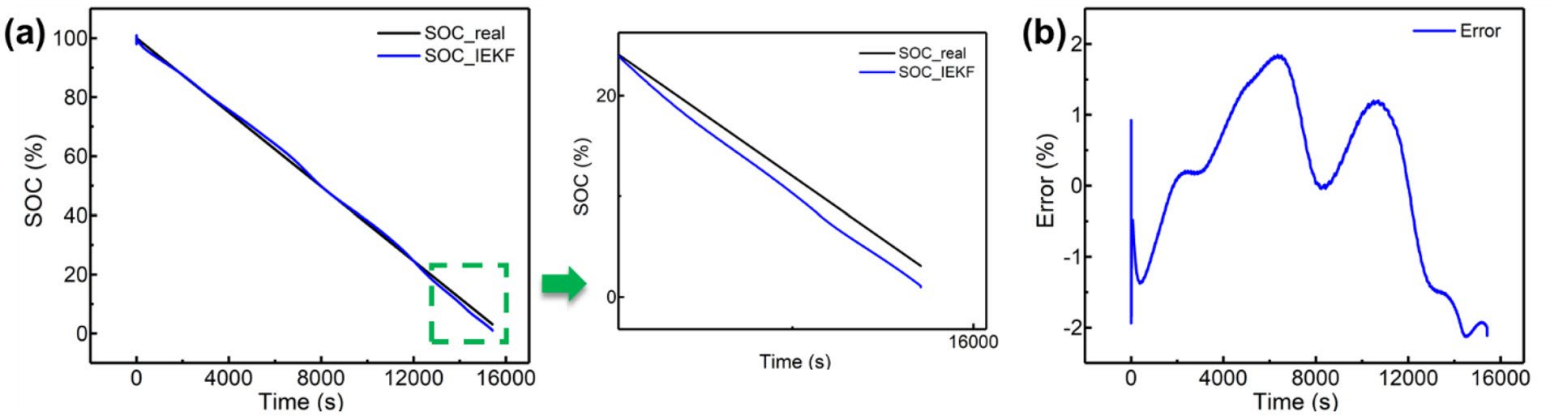
Result of SOC estimation under – 10 ℃. **(a)** The SOC estimation and max error is under 3%; **(b)** error analysis.

As shown in Fig. 8, the method is validated under low-temperature condition. The test is carried out at − 10 ℃ and 0.2 ℃ discharging until reaching cutoff voltage of 2.5 V. The maximum error under − 10 ℃ is 2.27%, indicating a satisfactory precision based on IEKF method.

**Figure 9:**
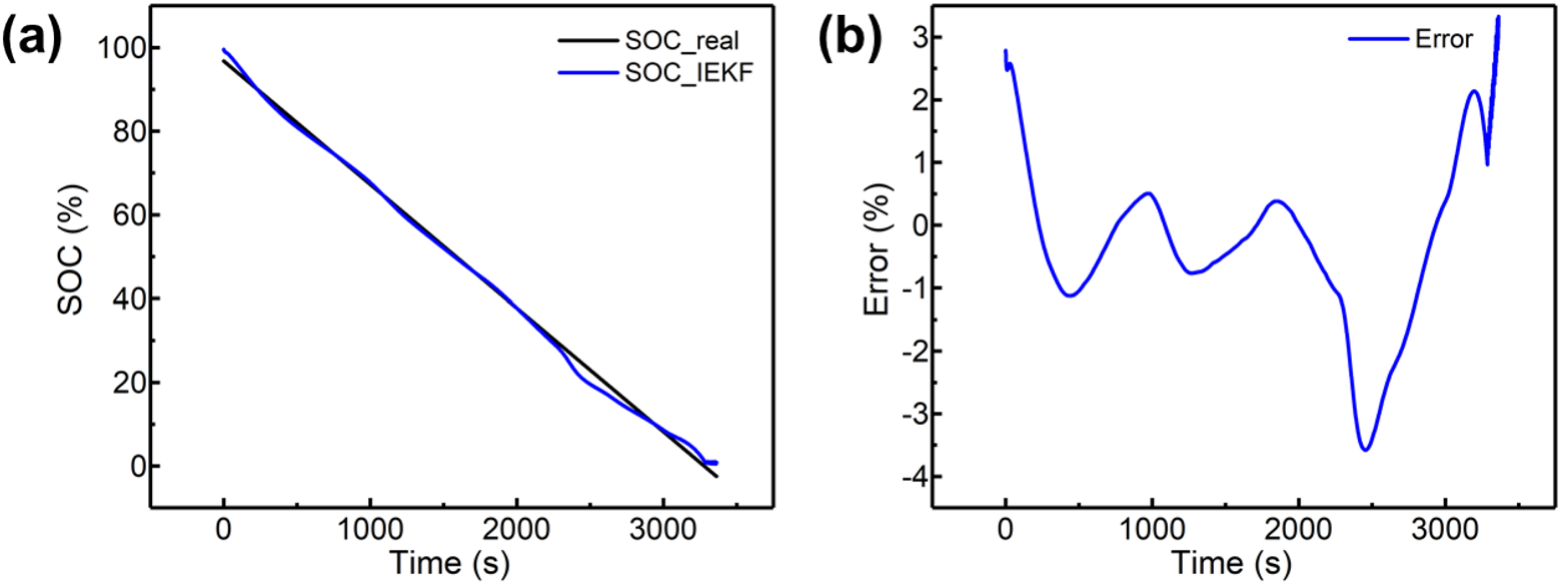
Result of SOC estimation based on lithium iron phosphate battery. **(a)** SOC estimation result; **(b)** error analysis.

Considering the various battery types operating on electric vehicles, the lithium iron phosphate (LFP) battery is selected as another typical example to validate the effectiveness of proposed method. Herein, a 1.5 AH LFP battery is operated at 1C discharging test and 25 ℃. The model of used battery is LR1865EH, and the tested result is presented in Fig. 9. The maximum error applying proposed method is under 3%, indicating the potential application of the proposed method for various battery types.

### Validation of convergence

The convergence of the algorithm can describe the ability of the filtering system to converge back to the true value when the system has seriously deviated. The quantification can be expressed as the time needed for the system to converge back to the true value.

**Figure 10:**
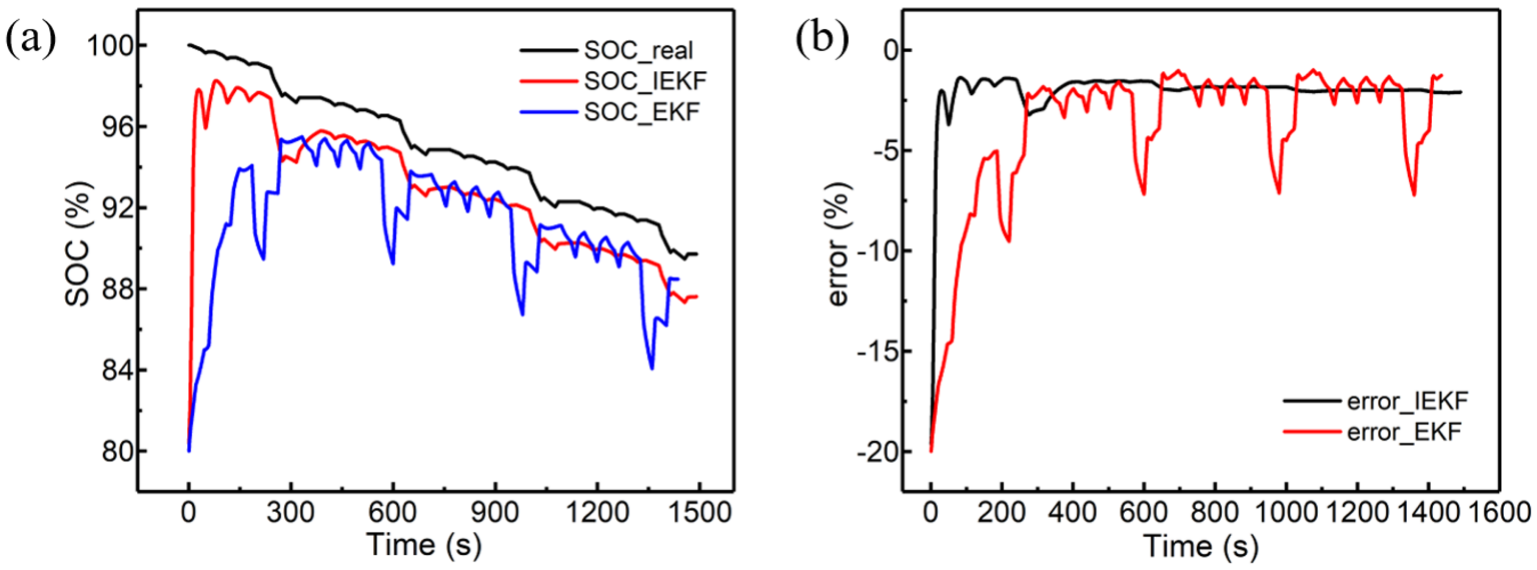
Result of convergence experiment: **(a)** applying a 20% deviation of initialization and the convergence of SOC; **(b)** error analysis.

Table 8 depicts the ability of system convergence under different degrees of deviation. Due to the use of the fading filter and adaptive noise, the convergence speed is significantly increased compared to the classical EKF algorithm. Furthermore, Fig. 10 illustrates the results of convergence when there is a 20% absolute deviation during SOC initialization.

**Table 8:** Convergence speed with different deviation.

`Deviation of absolute initialization	20%	40%	60%	80%	100%
EKF/s	198	243	337	364	470
IEKF/s	95	155	253	259	307

### Analysis of robustness

Robustness of the algorithm can be characterized as the ability to maintain true values when the system is disturbed. Since the state monitoring capability of the EV battery pack is limited in actual working conditions, the accumulated error caused by the current sensor is difficult to eliminate. Therefore, if the system samples contain the deviation and variance of the real current, the IEKF algorithm can trace the true value. Benefiting from the noise adaptive method, the IEKF algorithm has better robustness than the AHC.

In addition to the accuracy of the current sensor, the battery capacity (estimated SOC) is also greatly affected. The capacity is affected by many factors such as the ambient temperature and degradation of capacity. Table 9 lists the capacity test results of the batteries used in this paper at different ambient temperatures. It can be found in Table 9 that the nominal capacity of the battery is significantly different at various temperatures. Therefore, it is difficult to ensure that the battery capacity obtained by the AHC method is accurate for the actual usage of the battery pack. As a result, the algorithm robustness analysis should consider the battery capacity deviation problem.

**Table 9:** Battery capacity in different temperature.

Temperature/∘C	− 10		0	25	45
Capacity/Ah	111.15		115.53	129.12	135.78

**Figure 11:**
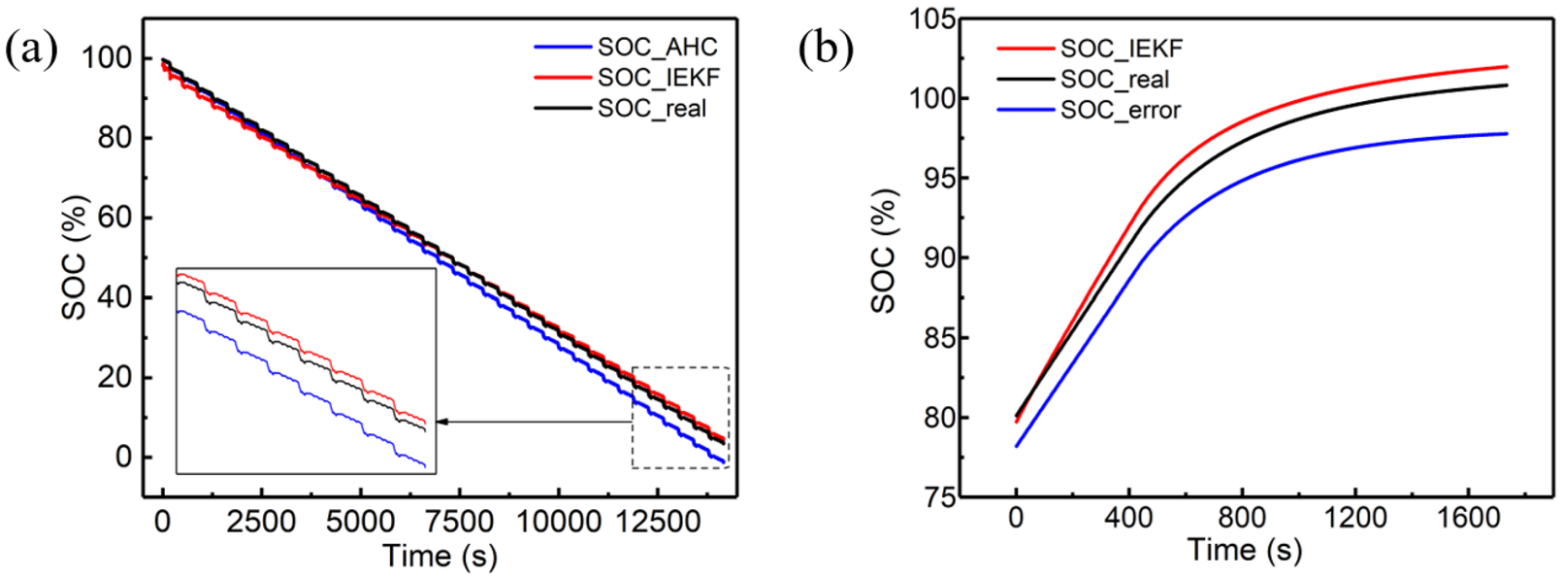
Results of robustness validation: **(a)** the result of mismatched capacity, **(b)** the result of deviation for current sensor.

Simulations of different capacities when calculating SOC under DST conditions are carried out. The results of capacity deviation and noise interference of the sensor are shown in Fig. 11. According to Fig. 11 and Table 10, the IEKF can tackle the mismatched capacity well compared to the AHC, by evaluating the error at the end of discharge, which indicates that IEKF has better robustness if the parameters of system are mismatched.

**Table 10:** SOC precision comparation between AH-integrity and IEKF.

Mismatched capacity	111.15	115.53	129.12	135.78
Error of AHC	16.54%	12.21%	0	4.65%
Error of IEKF	1.02%	1.33%	1.14%	1.25%

### Analysis of calculation efficiency

BMSs in EVs are always responsible for important tasks such as state monitoring, system protection, charging control and communicating with other electrical systems. Considering the limited computing ability of embedded systems in EVs, the calculation efficiency of the online algorithm is crucial. Therefore, we simulate the algorithm in MATLAB and verify the computational efficiency within the time span of the simulation. The results are shown in Table 11. Compared to traditional EKF, the IEKF with online parameter and L-N filter has similar calculation efficiency. The IEKF with online parameter and L-N filter is much more efficient than IEKF where the parameters are relying on central difference method.

**Table 11:** Comparation of calculation efficiency.

Working condition Computation time	CC discharge	CCCV charge	Pulse discharge	DST
IEKF with online parameter and LN filter/s	6.345	7.716	67.715	22.599
IEKF relying on central difference method/s	11.206	8.858	84.704	33.05
Traditional EKF/s	6.182	7.663	47.625	20.99

## Conclusion

An IEKF associated with online parameter identification is used to investigate the SOC estimation of LIBs for automotive applications. The algorithm highlights the fact that the model parameters and system noises need to be adapted to the inaccurate current/voltage measurement or other various environment noise interference. Simulations show excellent agreement with experimental data under different working conditions, and the maximum error is found to be 2.94% under DST conditions. Furthermore, the robustness and convergence are analyzed, showing that the IEKF has better performance than traditional EKF.

Noise interference is random and chaotic under practical operating conditions, which will affect the actual capacity of the cell and increase the difficulty of state estimation. The simulation results show that the maximum error of AHC for SOC estimation in distorted temperature is 16.54%, while the error of IEKF method is only 1.19%. Additionally, when there is a sensor error, AHC integrity has a significant deviation in the estimation due to its weak robustness, while the IEKF has much better accuracy. Moreover, simulation results show that IEKF has a higher convergence speed when dealing with mismatching initialization.

Although the proposed modelling framework and experiment only considers a single cell, the proposed algorithm can also be applied to further evaluate battery packs under varying operating conditions. In the future, temperature prediction and state of health estimation will also be considered in modelling and experimental work to further improve the method (Supplementary Information).

## Supplementary Information

Supplementary Information
