# Supplementary material for: A parameter adaptive method for state of charge estimation of lithium-ion batteries with an improved extended Kalman filter
Source: Sci Rep. 2021 Mar 11;11:5805. doi: 10.1038/s41598-021-84729-1 (PMC7952569; doi:10.1038/s41598-021-84729-1)
Supplement: Supplementary file 1 — Supplementary Information [file 41598_2021_84729_MOESM1_ESM.docx]

Supporting Information

A Parameter Adaptive Method for State Of Charge Estimation of Lithium-ion Batteries with An Improved Extended Kalman Filter

Shichun Yang^a^, Yang Hua^a^, Sida Zhou^a^,Xinan Zhou^a^, Xinhua Liu^a,^*, Yuwei Pan^a^, Heping Ling^b^, Billy Wu^c^

^a^School of Transportation Science and engineering, Beihang University, Beijing, China

^b^BYD Auto Industry Co., Ltd., Shenzhen, 518118, China

^c^Dyson School of Design Engineering, Imperial College London, London, UK

Corresponding author: liuxinhua19@buaa.edu.cn

1 Low-temperature experiment

A prismatic battery with 130 AH is selected for experimenting the characteristics under low temperature in the environmental chamber. Herein, the temperature is set as -10 ℃, and the battery is rested for more than 1 hour to achieve heat exchange balance between with environment. The characteristic of tested battery is presented as follows:

| **Serial numbers** | **Parameter item** | **Parameter value** | **Unit** |
| --- | --- | --- | --- |
| **1** | Capacity | 130 | Ah |
| **2** | Normal voltage | 3.6 | V |
| **3** | Max voltage | 4.2 | V |
| **4** | Min voltage | 2.5 | V |
| **5** | Max discharging current | 300 | A |
| **6** | Max charging current | 250 | A |
| **7** | Discharging/charging temperature range | -20~50 | ℃ |
| **8** | Cathode material | NCM |  |
| **9** | Anode material | Graphite |  |

The experiment is carried out with fully rested battery, and operational condition is 0.2C discharging. The battery will be fully charged until reaching the cutoff voltage, and then the battery will be rested for 1 hour. After the relaxation, the battery will be discharged with 0.2C current to the cutoff voltage. Both current and voltage will be observed, and the capacity and SOC will be estimated based on experiments.

1. LFP cell experiment

A 1.5 Ah cylinder battery with lithium iron phosphate (LFP) cathode is selected for experimenting the characteristics for investigating the generalization of proposed method. Considering the difficult issue for LTP battery SOC estimation owing to the wide voltage plat, thus the LTP battery is selected for research and the characteristic of tested battery is presented as follows:

| **Serial numbers** | **Parameter item** | **Parameter value** | **Unit** |
| --- | --- | --- | --- |
| **1** | Capacity | 1.5 | Ah |
| **2** | Normal voltage | 3.2 | V |
| **3** | Max voltage | 3.65 | V |
| **4** | Min voltage | 2.5 | V |
| **5** | Max discharging current | 4.5 | A |
| **6** | Max charging current | 1.5 | A |
| **7** | Discharging/charging temperature range | -20~45 | ℃ |
| **8** | Cathode material | LFP |  |
| **9** | Anode material | Graphite |  |

Similar with low-temperature experiment, the LFP battery experiment is carried out as fully charging, resting and fully discharging. However, the experiment is carried out at 25℃, and the battery has been set as heat exchange balance.
